# Fixed-Bed Modification of Zeolitic Tuffs and Their Application for Cr(VI) Removal

**DOI:** 10.3390/ma14227061

**Published:** 2021-11-21

**Authors:** Jolanta Karolina Warchoł, Paulina Sobolewska, Włodzimierz Tylus, Roman Petrus

**Affiliations:** 1 Department of Advance Material Technologies, Faculty of Chemistry, Wrocław University of Science and Technology, C-6 Building, 50-372 Wrocław, Poland; wlodzimierz.tylus@pwr.edu.pl; 2 Department of Water Purification and Protection, Rzeszów University of Technology, 35-959 Rzeszów, Poland; psobolew@prz.edu.pl; 3 Department of Chemical Engineering, Rzeszów University of Technology, 35-959 Rzeszów, Poland; ichrp@prz.edu.pl

**Keywords:** clinoptilolite, chabazite, HDTMA-Br, fixed-bed column modification, Cr(VI) removal

## Abstract

Natural clinoptilolite tuff (CL) and chabazite-clinoptilolite tuff (CH) were modified in fixed-bed column by immobilization of hexadecyltrimethylammonium bromide (HDTMA-Br), then investigated as a sorbent for inorganic anions of Cr(VI). The proposed modification technique combined with surfactant solution batching allows minimizing the surfactant loses through foaming and crystallization and creation of stable organic coverage. The HDTMA loading depended on the mineral composition of the zeolitic tuff, the topology of its external surface, and process conditions. The maximum surface coverage was obtained by gradually dosing surfactant solution in the smallest volume of batches and corresponded up to 100% and 182% of external cation exchange capacity (ECEC) for mono and double layer coverage, respectively. In case of mono layer coverage, modification proceeds until the exhaustion of surfactant in supply solution, while in the double layer one, until equilibrium of HDTMA concentration in both zeolitic and liquid phases was established. The efficiency of Cr(VI) uptake by prepared surface modified zeolites (SMZs) increased with increasing of HDTMA loading. In the case of mono layer SMZs, the capacities of CH-HDTMA and CL-HDTMA were 10.3 and 5.4 mg/g, respectively, while in the case of double layer SMZs, the amount of Cr uptake on CH-HDTMA and CL-HDTMA were 16.8 and 15 mg/g, respectively. Ion exchange is the predominant mechanism of Cr(VI) sorption but it takes place only if modification resulted in at least partial double layer coverage. The XPS analysis reveals Cr(VI) reduction to a less-toxic Cr(III) by the electron donating N-containing groups and by reaction with Fe^+2^ ions on the zeolite external surface.

## 1. Introduction

Cr(VI) is considered a highly toxic environmental pollutant mostly produced by industrial processes such as welding and pigment in paints and plastics. It is non-biodegradable, soluble in water in a wide pH range, and easily spread in the aquatic environment [1]. The conventional technique for Cr(VI) removal from a water stream is based on disposal-type processes (reduction–precipitation, liquid–solid transfer, precipitation–flocculation). Sorption-based techniques (ion-exchange, adsorption) are more attractive since they offer high efficiency of Cr(VI) removal and a possibility of its recovery by desorption and further sorbent reuse. Commercially available activated carbons [2] and synthetic resins [3] were presented as effective sorbents of Cr(VI) ions. Alas, for weak selectivity their practical application in treating metal-loaded sludge, soil remediation, re-use of industrial wastewaters, or building permeable barriers around landfills, spoil sites or communication routes, is economically unfavorable. Therefore, a growing exploitation to evaluate the feasibility and suitability of effective materials has been exerted [4]. Unfortunately, the alternative sorbents are not always available in amounts to meet commercial demand, have low sorption capacity in comparison to the commercial ones, and are not always suitable for regeneration and reuse.

Natural zeolites are abundantly available crystalline alumino-silicate minerals with 3D structure [5]. The isomorphic substitution of Si^4+^ by Al^3+^ creates its negatively charged framework structure accomplished by loosely bonded cations (e.g., Na^+^, K^+^, Mg^2+^, Ca^2+^) that maintain the network neutrality and are easily exchangeable with other inorganic cations [6]. The sum of the exchangeable cations attached to the sorption sites, held on the total specific surface area (m^2^/g), is CEC (meq/g). A part of CEC limited to the exchangeable cations localized on the external zeolitic surface is ECEC (meq/g). Both values (CEC and ECEC) depend on the content and properties of both zeolitic mineral and tuff impurities (e.g., micro-texture, crystallinity, chemical composition).

Considerable research over the past two decades identified that modification of natural zeolites with organic amines can provide an affinity for Cr(VI) oxianions [7]. There is a broad range of organic molecules applied for zeolite modification (Table S1), among which the HDTMA modified zeolites are the most commonly studied for Cr(VI) sorption [8]. Because of steric effect, surfactants cannot diffuse into zeolite, thus their sorption is limited to the external zeolitic surface. The modification of natural zeolites proceeds through two stages: the first one is a monolayer formation through the ion exchange and electrostatic attraction of N-containing groups as well as the methyl groups in the nitrogen head with the zeolite surface. If surfactant concentration is below the critical micelles concentration (CMC), it is sorbed as monomers. Above CMC, surfactant is sorbed in a form of micelles or elongated clusters, which then reorganize to form monolayer or patchy-bilayer coating. It was estimated that organic micelles with an aggregation number of 95 after rearrangement cover about 83 nm^2^ of zeolite surface [9]. The second stage is a subsequent organization of a bilayer coating via hydrophobic interactions with HDTMA carbon tails. Theoretically, the complete bilayer coverage requires the ratio 2:1 of the amount of HDTMA sorbed to the ECEC of zeolite [10]. In practice, an adsorbed amount above 1.5:1 is hardly obtained [11]. This is a consequence of abundance of loosely bound HDTMAs which are easily detached from organo-zeolites [12]. The formatted double-layer organic coat is stabilized by monovalent anions (Cl^−^ or Br^−^), which can be exchanged with oxyanions of Cr(VI). This process is considered the prevailing mechanism of chromium uptake.

A batch condition is the standard way for studying HDTMA sorption onto natural zeolites. This technique, however, requires maintaining a sufficient contact time of solid and liquid phase, and generates unreacted surfactant solution. Moreover, taking into account that there is nonlinear correlation between efficiency of Cr removal and surfactant concentration and loading, it seems necessary to identify an optimal modification’s conditions which will benefit the maximum Cr(VI) uptake [5]. However, we applied column transport experiments to provide the zeolite modification in open and sealed circulation units. The objective of this research was to evaluate the impact of flow rate of HDTMA-Br solutions and the frequency of its portioning on the modification efficiency. Other factors under investigation were the type of the modified mineral and the concentration of HDTMA-Br solution. Another aim of the research was to investigate the mechanism of both zeolite modification and Cr(VI) sorption onto mono- and double-layer. It was done by instrumental techniques (SEM, FT-IR, XPS) and by modeling of sorption equilibrium. The overriding goal of the research was to obtain cheap materials with high sorption affinity for Cr(VI) oxyanions, which could potentially be applied for creation of permeable reactive barriers in the area of waste landfills [13]. For this, the capability of SMEs for chromium uptake from multi-component real wastewater was tested to identify its selectivity towards Cr(VI) ions over other inorganic contaminates.

## 2. Materials and Methods

### 2.1. Reagents

All used chemicals were of analytical grade: HDTMA (Mw: 364.4 g mol^−1^, Merck), NaCl (0.99, Merck), AgNO_3_ (0.99, Merck), tetraethylammonium bromide (TMA, 0.98, Sigma-Aldrich), ICP Cr standard (Fluka), K_2_CrO_4_ (Sigma-Aldrich), ethanol (0.96, Merck), and HNO_3_ (0.69, Merck). Working solutions were prepared by diluting the stock solutions with deionized water (0.065 µS cm^−1^, SolPure 7, POL-Lab, Wilkowice, Poland).

### 2.2. Used Sorbents and Their Characteristics

The sample of clinoptilolite (CL) volcanic rock came from deposit located in Nižný Hrabovec, Slovakia, while the sample of chabazite-clinoptilolite (CH) from deposit in Winston, USA. Both minerals are characterized as microporous aluminosilicates. Chabazite framework structure comprises six eight-member rings of 3.8 × 3.8 Å opening into large ellipsoidal cavities of 6.7 × 10 Å (chabazite cage) [14], while monoclinic crystal structures of clinoptilolite contain three sets of intersecting channels: ten-membered rings of 3.1 × 7.5 Å confined by eight-membered rings of 3.6 × 4.6 Å or of 2.8 × 4.7 Å [15]. The milled zeolitic crushed rock aggregates (jar mill, LMW-S, TESTCHEM) were sieved (screen, Haver and Boecker) to 0.2–0.5 mm diameter particles, washed with deionized water, and dried at 110 °C (laboratory dryer, POL-EKO) for 24 h.

The identification of the crystalline species was done by X-ray powder diffraction (XRPD) analysis (model Kristalloflex 4H, Siemens), with CuKα = 0.145 nm radiation, operating at 30 kV and 25 mA. The data identified the following mineralogical composition of the zeolitic materials:

CL: 74% clinoptilolite, 11% cristobalite, 6% plagioclase, 4% illite and smectite, 3% tridymite, 1% kaolinite, and 1% quartz;
CH: 54% chabazite, 36% clinoptilolite, 5% quartz, 5% unidentified.

The chemical composition of both zeolitic materials was identified by wavelength dispersive X-ray fluorescence (WDXRF) technique by using AxiosmAX (PANalytical) analyzer. The elementary analysis was based on the Omnian program. The *n*Si/*n*Al ration, calculated on the data depicted in Table 1, equals 5.11 and 3.56 for CL and CH, respectively. The identified orders of exchangeable cations are as follows:

For CL: K^+^ > Ca^2+^ > Fe^2+^ > Mg^2+^ > Na^+^ > Ti^4+^ > Ba^2+^ > Sr^2+^,
For CH: Na^+^ > Fe^2+^ > Ca^2+^ > K^+^ > Mg^2+^ > Th^4+^.

The textural parameters of the zeolite samples were determined by using a Micromeritics, ASAP 2420 (Micromeritics). Nitrogen adsorption isotherms were obtained at liquid N_2_ temperature. To calculate the specific surface area (*A*_BET_), the Brunauer–Emmett–Teller (BET) equation was applied. The total pore volume (*V*_T_) was then evaluated by converting the volume of nitrogen adsorbed at *p*/*ps* ≈ 0.98 to the volume of liquid adsorbate. The micropore surface area (*A*_mic_) was determined using De Boer’s *t*-plot method. It should be noted that the N_2_ molecule (diameter 0.37 nm) is too large to penetrate micropores of the zeolites occupied by exchangeable cations and water molecules [16]. However, the obtained value of textural parameters should be treated as reference values (Table 2).

The CEC of the zeolites was determined by contacting the Na-form of zeolite with 1 M CaCl_2_ for 24 h and analyzed for Na^+^ content by AAS (SpectrAA 880, Varian). The ECEC of zeolites was determined by the adsorption of tetraethylammonium bromide (TMA) on the Na-zeolites. For this purpose, 1 g of Na-form of zeolite and 100 mL of deionized water were mixed with a magnetic stirrer for 3 h at 80 °C. Then, a portion of TMA (1g 100 mL^−1^) was added to the suspension at each 1.5 h time interval, until the total solution volume was 200 mL. The solution was decanted and analyzed for C content (TOC Sievers InnovOx, GE Analytical Instruments, USA). The amount of surfactant retained in material was calculated from a mass balance equation:

(1) $$q_{e}=V\cdot \left(C_{0}-C_{e}/m\right)$$

where *C*_0_ and *C*_e_ are the initial and equilibrium TMA concentrations in the solution (g L^−1^), respectively, *V* is the volume of TMA solution (L) and *m* is the sorbent dosage (g L^−1^). To confirm an accuracy of the ECEC measured, the remaining zeolites were washed with deionized water (80 °C) and 96% ethanol, dried (105 °C), and analyzed for N, C, and H content (CHNS Vario EL III, Elementar). The ECEC value was calculated from the difference in CHN content in raw and treated with TMA zeolite. Data obtained are presented in Table 3.

Zeta potential measurements were done using a Malvern Instruments Zetasizer Nano ZS by electroforetic light scattering technique. 

ATR-FTIR spectra were recorded on a FT-IR spectrometer (Nicolet 8700, Thermo Scientific) equipped with an ATR (single reflection type, Smart Orbit™ diamond). A diamond prism was used as the waveguide. The size of the IR beam was 2–3 μm and the incidence and reflection angles were both 45°.

XPS spectra were acquired with a SPECS PHOIBOS100 spectrometer with MgKα X-ray source. Kinetic energies of the photoelectrons were measured using a hemispherical electron analyzer working in the constant pass energy mode. The spectrometer was operated at 250 W for high resolution spectra. The base pressure in the UHV chamber was better than 5E-10 mbar. The analyses were performed for the powdered samples pressed into double adhesive copper tape. Binding energies (BEs) were referenced to 284.8 eV for C 1 s in C-C/CH bonds. Surface etching during XPS measurements was carried out by Ar^+^ sputtering with gentle beam energy of 1 keV and ion current density of 1.5 µA cm^−2^.

### 2.3. Zeolite Pre-Treatment

Pretreatment of the zeolites was performed contacting 50 g of the zeolitic material with 250 mL of 0.5 M NaCl solution at ambient temperature. The mixture was agitated (at 100 rpm) for 24 h in mechanic shaker (model WL-2000, WElectronic). Afterwards, the adsorbent was separated by decantation. The salt excess was removed by subsequent dialysis (cellulose membranes, Sigma–Aldrich) monitored by the Mohr (AgNO_3_) test. The obtained Na-form of CL-Na and CH-Na were dried at 105 °C for 24 h.

### 2.4. Zeolite Modification

The Na-zeolites (CL-Na and CH-Na) were modified with HDTMA-Br. Its mass, corresponding to a given zeolitic material ECEC, was calculated from the following equation:

(2) $$m_{\mathrm{HDTMA}}=\left(M/P\right)\cdot x\cdot \mathrm{ECEC}\cdot m$$

where *m* is zeolite mass (g), *M* and *P* are molar mass (g mmol^−1^) and purity of HDTMA-Br, respectively, and *x* is number of organic layers equivalent to 1.0 or 2.0 or 4.0 ECEC. The weighed HDTMA-Br mass was dissolved in a given volume of deionized water to obtain the initial surfactant concentration *C*_0 HDTMA_ = 525, 1050, 2100 mg HDTMA L^−1^, which in all cases > CMC. The modification process was conducted in a 10 mm (I.D.) borosilicate glass column (model LRC, PALL) of 10.5 cm height. The surfactant solution was kept in a temperature-controlled ultrasonic bath (Polsonic) at 20 or 40 °C to avoid crystallization. It was passed through the column in an upward direction using a peristaltic pump (model 70985-00, CAT). Figure S1 illustrates the experimental set implemented for both an open and a sealed circulation unit. In case of the open unit, the process was carried out until the concentration at the column outlet (*C*_out HDTMA_) was equal to the concentration at the column inlet (*C*_0 HDTMA_). For the applied flow rates *Q* = 0.2, 0.4, 2, and 4 mL min^−1^ the time after which *C*_out HDTMA_/*C*_0 HDTMA_ = 1 equals 45 h 50 min, 20 h 50 min, 1 h 55 min, and 1 h 15 min, respectively. Meanwhile, each subsequent 50 mL of the column effluent was subjected to TOC analysis. In the sealed circulation unit, the samples of effluent were taken after a time corresponding to 1.5, 2, 2.5, and 3 times of solution circulation through the fixed bed. Additionally, in this unit case, the volume of the surfactant solution circulated was gradually increased by the consecutive addition of HDTMA batch (*B*) at fixed time intervals. The influence of HDTMA concentration was determined by variable numbers of batches (15, 30, 60) with each batch totaling the same volume of surfactant solution (300 mL). To examine the effect of HDTMA mass, the number of batches was differentiated (*B* = 30, 60, 120 for *x* = 1.0, 2.0, 4.0 ECEC, respectively), maintaining constant both *C*_0 HDTMA_ and time interval between subsequent batches sampling. The conditions of zeolites modification are summarized in Figure S2. In both the sealed and open unit runs, the feeding of the column was continued until no identification of a change of HDTMA concentration in the outlet. The degree of surface covering (*DC,* multiples of ECEC) was calculated from the following equation:

(3) $$DC=x\cdot \;\left(C_{0\;\mathrm{HDTMA}}-C_{out\;\mathrm{HDTMA}}\right)/C_{0\;\mathrm{HDTMA}}$$

where *C*_out HDTMA_ is concentration of surfactant in the column outlet (mg L^−1^). The SMZs obtained were gently washed with deionized water (80 °C) until no Br^−^ was detected (the Mohr’s method). Part of the material modified with *x* = 1 ECEC was additionally washed with 96% ethanol [17]. Then, the SMZs were dried at 105 °C and identified as CL-HDTMA and CH-HDTMA.

### 2.5. Chromium Sorption on SMZ

#### 2.5.1. From Artificial Solutions

The equilibrium experiments were performed under batch conditions at ambient temperature. This trial included 29 vials, each contained 0.1 g SMZ and 10 mL of chromium solution ranged from 0.5 to 1500 mgCr(VI) L^−1^. For this, 5.6 g of K_2_Cr_2_O_7_ was dissolved in Milli-Qwater at pH_init_ 3.0 and then gradually diluted in deionized water at corresponding pH_init_ to obtain the desire chromium concentration. The pH_init_ is reported as the optimum pH value for the Cr(VI) sorption on SMZs [18]. The pH_init_ was adjusted by using 0.1 M HNO_3_ and measured using a standard pH meter (FE20-ATC/Five Easy Plus, Mettler Toledo), equipped with an electrode (LE438, Mettler Toledo) calibrated by using standard buffer solutions at pH_init_ 3.0 ± 0.01 (duracal buffer). The vials were shaken (100 rpm) for 5 h and leaved for further contact until *t*_c_ = 24 h. Afterwards, the solids were separated from the liquids by decantation. Supernatants were analyzed by ICP-OES (Integra XL, GBC Scientific Equipment) to evaluate the concentration of chromium (detection limit 0.1 g Cr L^−1^, *R*^2^ for the standard curve 0.999), and by pH measurements, to determine the pH_eq_. All experiments were performed in triplicate. The results obtained were given as mean values. The amount of Cr(VI) sorbed on the zeolitic materials was calculated from the mass balance equation (Equation (1)). The equilibrium data obtained were used to model the adsorption equilibrium by commonly used empirical models [12,18]:

(4) $$\mathrm{Freundlich model}:\;q_{e}=KC_{e}^{1/n}$$

(5) $$\mathrm{Langmuir model}:\;q_{e}=\frac{KC_{e}q_{m}}{1+KC_{e}}$$

(6) $$\mathrm{Bilangmuir}\;\mathrm{model}:\;q_{e}=\frac{K_{1}C_{e1}q_{m1}}{1+K_{1}C_{e1}+K_{2}C_{e2\;}}+\frac{K_{2}C_{e2}q_{m2}}{1+K_{1}C_{e1}+K_{2}C_{e2\;}}$$

where *q*_e_ (mg g^−1^) and *C*_e_ (mg L^−1^) are the equilibrium chromium concentrations in solid and liquid phase, respectively, *q*_m_ (mg g^−1^) is the maximum adsorption capacity, and *K* represents the equilibrium constant. Modeling calculations were conducted using the Maple program by means of a nonlinear regression method based on the Levenberg−Marquardt algorithm and by minimizing the sum of the squares of the error (SSE) function.

#### 2.5.2. From Real Wastewaters

The wastewater was collected from the municipal wastewater plant in Radomsko (Poland). The chemical composition of wastewaters was analyzed using total reflection XRF X-ray fluorescence spectrometer (S2 PICOFOX). No sludge or flocculent suspension was found in the wastewater. The sorption capacity of the newly prepared adsorbents, Na-zeolites (CL-Na and CH-K-Na) and SMZs (CL-HDTMA and CH-HDTMA) modified in sealed circulation unit (*Q* = 4 mL min^−1^, *B* = 60) with HDTMA (*x* = 1.0 and 2.0 ECEC), was evaluated using batch experiments. For this, 0.1 g of sorbent and 10 mL of raw sewage were placed in polyethylene falcons of 50 mL. The samples were shaken using a rotary shaker at 40 r min^−1^ for 24 h at ambient temperature. Then, the liquid phase was separated from the solid phase by decantation, and the pH value and the residual chemical species concentration were measured in the same way as before sorption.

## 3. Results and Discussion

### 3.1. Zeolites Modification

**Figure 1:**
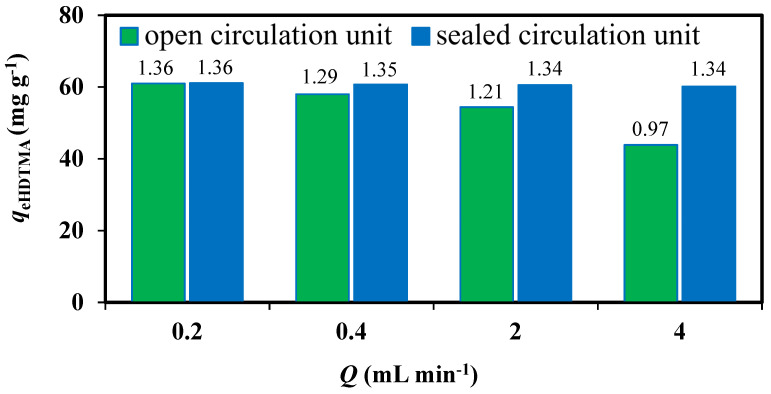
Influence of the type of circulation unit and surfactant solution flow rate on efficiency of CL modification. *DC* values over the bars. *C*_0 HDTMA_ = 1050 mg L^−1^, *T* = 25 °C, *x* = 2 ECEC.

**Figure 2:**
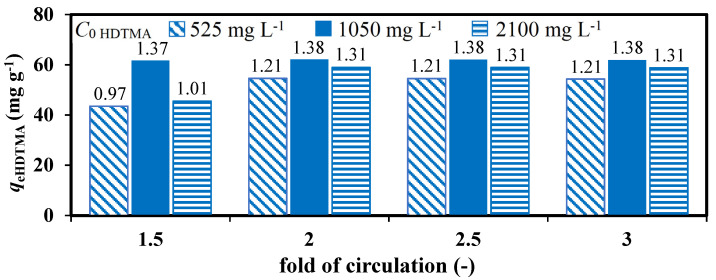
Impact of surfactant solution concentration and multiplicity of its circulation on efficiency of CL modification. *DC* values over the bars. *Q* = 4 mL min^−1^, *T* = 40 °C, *x* = 2 ECEC.

The influence of the surfactant solution flow rate on the modification efficiency was assessed for the open and sealed circulation units. The data depicted in Figure 1 show that the increase of the flow rate decreased the efficiency of modifications in the open cycle unit. This is closely related to the shorter contact time. Furthermore, there was a high risk of air sucking and foam formation. In the sealed circulation unit case, no change in modification efficiency over flow rate was observed. It is well known that the HDTMA concentration specifies the ratio of monomers to micelles in the solution, which always remains in dynamic equilibrium. At the beginning of the modification process, when the concentration of HDTMA > CMC, the micellar form of surfactant predominated in solution. At the same time, sodium cations were released due to the ongoing ion exchange reaction Na^+^/HDTMA^+^ (monolayer formation). Then, as time of circulation went on, the HDTMA concertation decreased. Both the change in HDTMA concentration and the presence of electrolytes influenced the stability of micelles, which was related to their aggregation number and consequently their size and shape [19]. It was identified by TOC analysis that after the time corresponding to the 2-fold sealing of the surfactant solution, *C*_e_ HDTMA (346 mg L^−1^) ≈ CMC (328 mg L^−1^). Then, the formed micelles had an elongated rather than spherical shape with an aggregation number limited to a few dozen monomers [20]. After sorption on the zeolite surface, they formed bimolecular layer by admicelle degradation [21]. However, the monomers’ contribution to reduction of intramicellar channels cannot be excluded. Their presence on the zeolite surface alongside micelles was confirmed by the DTG analysis [22]. Nevertheless, the obtained *DA* values (see the value over the bars Figure 1) were far below the expected, equivalent to *x* = 2.0 ECEC. Thus, in the next step, the influence of surfactant concentration was analyzed. The data depicted at Figure 2 were obtained by analysis of the effluent samples after different times of solution volume circulation in order to check the organic layer stability. The HDTMA mass used was the same in each surfactant solution regardless of the concentration. The temperature of HDTMA solutions was adjusted at 40 °C to increase the degree of micelles dissociation (averaging 0.002 per 1 °C) [23]. It resulted in a slightly bigger HDTMA adsorption than at 25 °C (Figure 1 bar for *Q* = 4 cm^3^ min^−1^). As can be seen, regardless of the number of folds of the solution circulation, the modification efficiency obtained for *C*_0_ = 1050 mg L^−1^ was greater than that for C_0_ = 525 mg L^−1^. It is obvious that the proportion of micelles to monomers in the solutions of higher concentration was greater than in lower ones. Micelles are less hydrated than monomers, and therefore more attracted to the oppositely charged surface of the zeolite. However, further increase of the surfactant concentration (*C*_0_ = 2100 mg L^−1^) resulted in the reduction of the modification efficiency. This stems from the fact that the repulsive forces between the polar heads begin to dominate in high enough concentrated solution. This weakened the interactions between the surfactant heads and the zeolite surface until the point when they became comparable to the interaction forces between the hydrophobic chains. Accordingly, the proportion of micellization in the solution decreased and the proportion of micellar aggregation near the zeolite surface increased. Surface micelles can be easily detached from the polar zeolite surface under the effect of filtration [5]. This causes the reduction of process efficiency (*DA* value) observed for the highest concentrated solution which is in line with a non-linear correlation between an initial *C*_0_ = 525, 1050, and 2100 mg L^−1^ and an equilibrium, remained in solution, HDTMA concentration *C*_e_ = 208, 327, and 729 mg L^−1^, respectively. It should be also emphasized that no foaming or surfactant crystallization was observed for modification in sealed circulation unit, even for the highest HDTMA concentration. During subsequent folding of given batch circulation, an attractive force between the polar heads of the surfactant and the zeolite surface again began to dominate. Its outcome was a creation of stable organic layer, seen on the graph as unchangeable *DA*s after 2- and higher fold of solution circulation.

**Figure 3:**
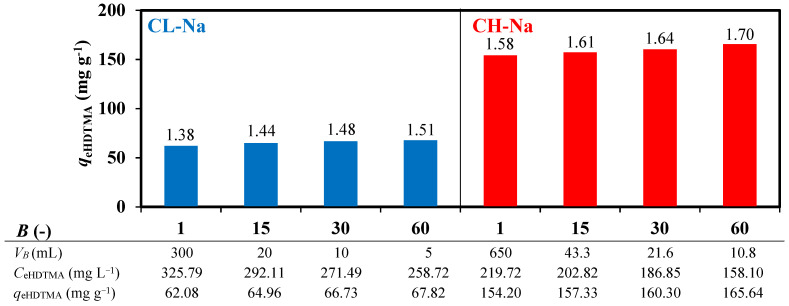
Impact of surfactant solution batching (*B*) on efficiency of zeolite modification in sealing circulation unit. *DC* values over the bars, *Q* = 4 mL min^−1^, *C*_0 HDTMA_ = 1050 mg L^−1^, *T* = 40 °C, *x* = 2.0 ECEC.

Figure 3 reports the influence of the surfactant solution batching on the modification efficiency of two zeolitic materials (CL-Na and CH-K). It should be remarked that regardless of numbers of batches, the time interval between subsequent batches was set to ensure 2.5-fold solution circulation, with total duration of sampling kept constant for a given zeolitic material. The data depicted on Figure 3 indicate that increasing the number of batches, understood as the reduction of single batch volume, increased the modification efficiency (*DC*). Each batch sampling resulted in decreasing the HDTMA concentration (from *C*_0 HDTMA_ > CMC to *C*_e HDTMA_ < CMC) along with circulation fold. Furthermore, each subsequent batch became more diluted at the moment it reached the fixed bed (from *C*_0 HDTMA_ > CMC to *C*_0 HDTMA_ < CMC), as the volume of solution in the sealed unit increased. Both ongoing changes influenced the micelle/monomer ration as well as micelle size. 

At the beginning of the process, during the time of single batch circulation, the most rapidly sorbed were micelles with the highest aggregation number as possessing the highest positive charge. They rearrange on the negative zeolite surface as space and charge permit forming loosely packed bilayer [9]. Then, smaller sized micelles and monomers could also participate in the increase of density of packed bilayer. With the batching advancement, the surface coverage increased causing hydrophobicity of the surface and repulsion of hydrophilic micelles. Simultaneously, as each subsequent batch became more diluted at the moment it reached the fixed bed, the surfactant solution contained more monomers. Because of a small size, they could migrate through intramicellar channels to the hydrophobic surface further increasing density of the organic coating. During the whole batching process, both sorption equilibrium and dynamic equilibrium between micelles and monomers were reached before each subsequent batch addition. The equilibria concentrations changed along with the process advancement. Hence, the higher number of batches resulted in shifting equilibrium concentration *q*_e HDTMA_ to higher values, while *C*_e HDTMA_ to lower one. During the batching, no foaming of surfactant in the experimental setup was observed.

Furthermore, beside process conditions and surfactant properties, the zeolitic material properties also influenced the degree of zeolite coverage. The comparison of the data depicted in Figure 3 identified that the amount of sorbed HDTMA on CH-Na material was about 2.5 times greater than on CL-Na. The apparent difference directly relates to the zeolites’ mineralogical composition, structure, and size of the external surface. CH tuff has a 3-fold larger external surface (*A*_BET_, Table 2) and over 2-fold higher external cation exchange capacity than CL tuff (ECEC, Table 3). The porosity of natural zeolites is, to a large extent, attributed to the micropores that diameters are too small to be penetrated by the polar head of surfactant (0.694 nm). Thus, total pore volume (*V*_T_, Table 2) did not have any effect over the HDTMA adsorption [24].

**Figure 4:**
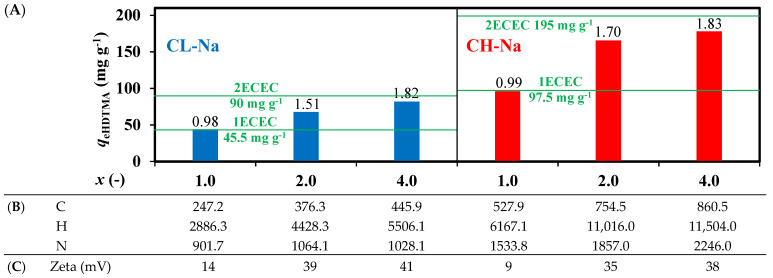
(**A**) Impact of HDTMA mass on efficiency of zeolite. *DC* values over the bars, (**B**) CHN analysis (mg 100 g^−1^ of the SMZ), (**C**) Zeta potential. *Q* = 4 mL min^−1^, *C*_0 HDTMA_ = 1050 mg L^−1^, *T* = 40 °C.

The data depicted in Figure 4 were obtained for the studies in which the HDTMA-Br mass corresponding to *x* · ECEC was differentiated but keeping the same concertation ***C***_0 HDTMA_. Regardless of the total number of batches (each *V_B_* = 5 mL), the time interval between two subsequent batches was constant. Thus, with increasing the number of batch (for *x* = 1.0, 2.0, 4.0 ECEC, *B* = 30, 60, 120, respectively) the total duration of batching increased. The results in Figure 4A show that for modification equivalent to *x* = 1.0, the efficiency of HDTMA-Br extraction from circulated solution was 98% and 99% for CL-Na and CH-Na, respectively. Further increase of HDTMA mass did not result in *DC* = 2.0 (200% of coverage) neither for *x* = 2 nor *x* = 4.0. For *x* = 2.0, the amount of surfactant remaining in solution was 25% and 15% for CL-HDTMA and CH-HDTMA, respectively, while for *x* = 4.0 ECEC, regardless of SMZ, up to 55%. Based on the above, it is evident that the binding forces of the single layer (polar head to polar surface of zeolite) are much higher than the interaction forces in the second layer (hydrophobic interactions between alkyl chains) [25]. In the latter case, there was a reversible adsorption until the equilibrium concentration of HDTMA in both phases was attained.

The efficiency of modification was further confirmed by CHN analysis of SMZs (Figure 4B). The comparison of *DC*s depicted in (Figure 4A) showed high similarity in relation to analytical methods applied to direct measurement of organic coverage as HDTMA mass on the zeolite surface and indirect measurement of remaining organics as an equilibrium concentration in liquid phase (TOC analysis, Figure 4A). As it stems from the table (Figure 4B), regardless of zeolitic material, the content of C, H, and N on SMZ modified with *x* = 1.0 ECEC was lower than 2-times of corresponding values obtained for *x* = 2.0 and 4.0 ECEC. An increase of surface coverage with increase of *x* was further confirmed by Zeta potential measurements (Figure 4C). The Na-zeolites have a negative surface charge, −18 mV and −24 mV for CL-Na and CH-Na, respectively. The evident reversal in surface charge appears after zeolite modification. Nevertheless, as was also observed by other authors, there was no linear correlation between *x* and zeta values [26]. Still, the highest value of zeta obtained for *x* = 4.0 ECEC identify that the organic layers obtained for *x* = 2.0 ECEC were not fully completed. 

**Figure 5:**
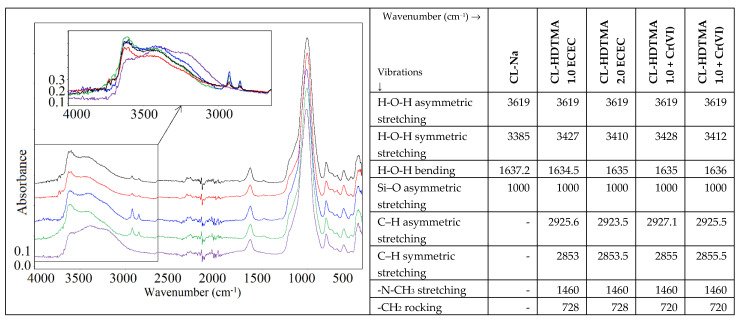
FTIR spectra of Na-form zeolite and SMZs before and after Cr(VI) sorption.

Figure 5 displays the ATR spectra obtained before and after zeolite modification together with assignment of respective bands. The vibration bands depicted can be grouped into those associated with zeolite and those with organic layer. Two evident differences between the spectra of unmodified and modified zeolite consist in the position of the band responsible for the methylene stretching and scissoring vibration. Integral intensity of –CH_2_– group together with slight shift of the C–H asymmetric stretching from 2925.6 cm^−1^ for HDTMA loading *x* = 1.0 ECEC to 2923.5 cm^−1^ for *x* = 2.0 ECEC (*DC* = 1.5) revealed an increase in the number of *trans* (order) conformers [27]. The shift in the direction of lower wavenumbers upon with increase of organic loading was also noticed by other authors [28]. In turn, the C–H symmetric stretching vibration preserves an unchanged position. The band at 1460 cm^−1^ is associated with N-CH_3_ stretching of the cationic surfactant [29].

To have deeper insight into HDTMA loading, XPS analysis was carried out on the zeolitic surface. It should be noted that the XPS technique is very surface sensitive, the sampling depth does not exceed the value of 3λ (inelastic mean free pathway for electrons, IMFP), and 66% of the analytical information comes from a layer only 1 λ thick. In the case of organic compounds, such as HDTMA, exemplary values of 1 λ for Br 3d or Cr 2p photoelectrons are approximately 3 and 2 nm, respectively. XPS is therefore a particularly well-suited technique for testing surface-modified zeolites with an organic layer thickness below 2–3 nm. The disadvantage of this technique is that in computational methods, it is assumed that the composition is homogeneous within the sampling depth, which is usually not true. The data in Table 4 represent the elemental composition of analyzed surface ‘as-received’ and after mild etching by Ar^+^ beam (1 keV, 2.5 µA cm^−2^). Despite the number of organic layers, all analyzed SMZs beside components of zeolitic bed (Si, Al, O) contained N and C. 

**Figure 6:**
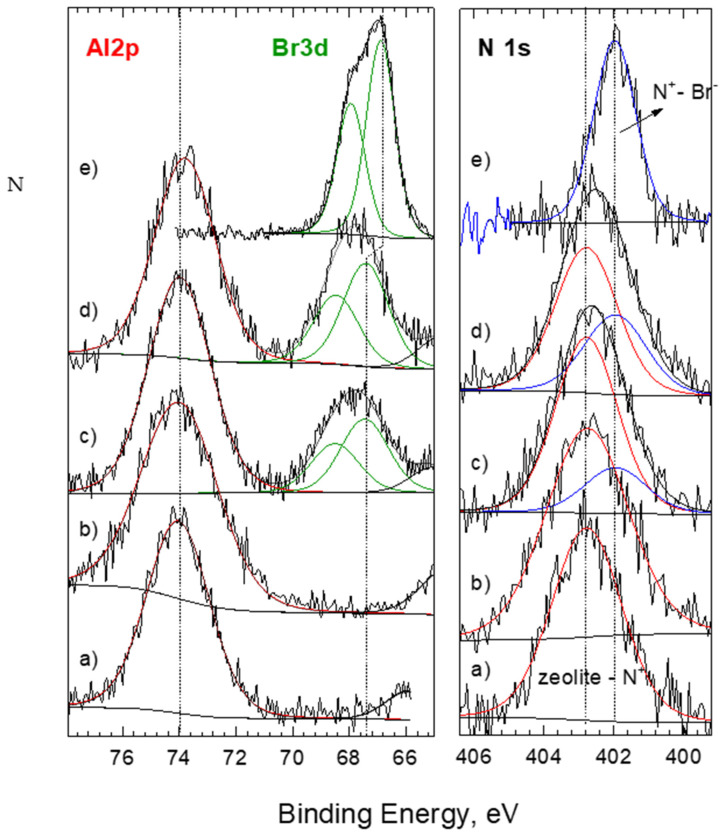
XPS N 1s and Al2p&Br3d spectra for (**a**) CH-HDTMA 1.0 ECEC, (**b**) CL-HDTMA 1.0 ECEC, (**c**) CH-HDTMA 2.0 ECEC, (**d**) CL-HDTMA 2.0 ECEC, and (**e**) HDTMA as a reference.

In the case of a single organic layer SMZ, the N and C content was 1.1 and 34.2% at for CL-HDTMA, and 0.9 and 25.1% at for CH-HDTMA, respectively. For both SMZs, the N:C ratio was 0.03, which corresponds to the value obtained for the pure HDTMA. This demonstrates that the N and C presence on the zeolite surface originated from the modification process. Meaningfully, there was no presence of Br. The analysis of the dynamics of changes in the chemical composition allowed for a qualitative assessment of the distribution of elements in the cross-section of the modified single layer. A diversified decrease in N:Si and N:Al ratios was observed along with Ar^+^ sputtering, which indicates that some of the immobilized HDTMA was trapped in the pores and cavities of zeolitic material. To be more precise, lower decrease observed in the case of CH (25%) compared to CL (57%) resulted from the more flattened surface of the latter. The lack of Br and the evident shift of N 1s binding energy (for pure HDTMA, Figure 6e) to 402.8 eV (for both CL- and CH-HDTMA, Figure 6a,b) affirm that HDTMA binding to the zeolitic surface occurs through N^+^.

In the case of a double organic layer SMZ, the absolute values of C and N were higher than for materials loaded with *x* = 1.0 ECEC. Nevertheless, the difference in N content (as received) between mono and double layers equaled 61% and 31% for CL-HDTMA and CH-HDTMA, respectively. This confirms a partial HDTMA capture in external pores and caves on external CH zeolite surface, which made it difficult for a double layer to be formed there [22]. A decrease in relative N/Si and N/Al ratios along with Ar^+^ etching revealed a heterogeneous chemical composition in sub-nanometric dimensions. Furthermore, the observed decrease was higher for CL-HDTMA (39%) than for CH-HDTMA (19%), resulting from more regular (sandwich) organic double layers formed on flat surface of the CL-HDTMA. Figure 6c,d confirms Br presence on the double organic layer SMZs. For both materials, Br 3d5/2 bond energies were estimated at 67.4 eV, which is very close to pure HDTMA (66.9 eV). This supports the concept that a part of HDTMA molecules is chemically unbounded with the zeolitic surface [30]. In the case of CL-HDTMA, it was estimated at 37% of the total amount of N, and in the case of K-HDTMA at 20.7%. This further confirms a smaller fraction of the second layer on the CH-HDTMA compared to CL-HDTMA.

### 3.2. Cr(VI) Sorption on SMZ

**Figure 7:**
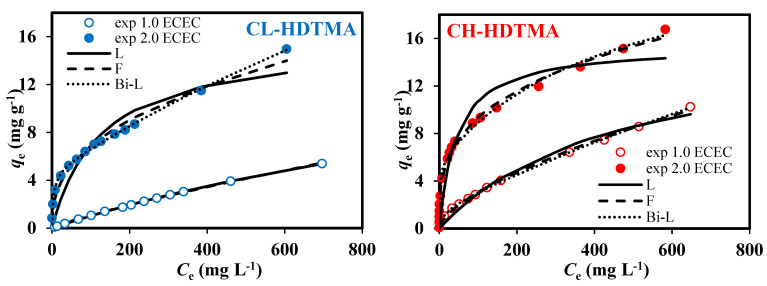
Comparison of equilibrium isotherms and modeling curves obtained for Cr(VI) sorption on SMZs.

**Figure 8:**
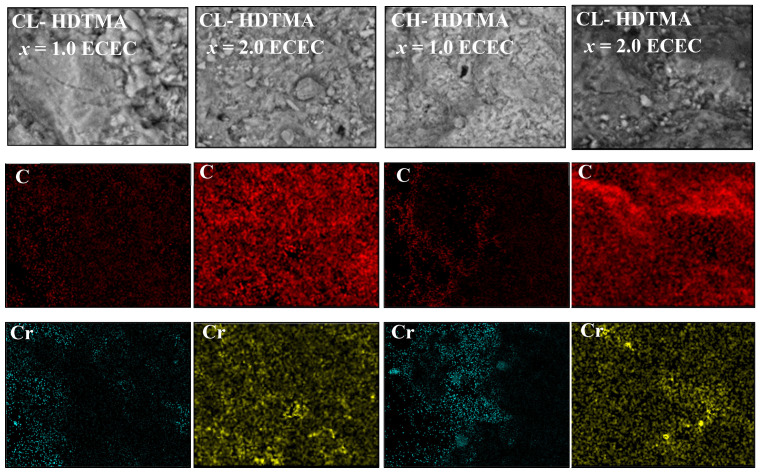
SEM-EDX C and Cr element mapping images of SMZ before and after Cr(VI) sorption.

The data obtained from sorption experiments (Figure 7) identify Cr(VI) presence on both mono- and double-layer SMZs. This was further confirmed by the SEM/EDS examinations of the Cr-loaded SMZs (Figure 8). Uneven Cr distribution (mapping images) on the modified surface corresponds to the irregular distribution of C atoms observed on the SMZs. An upward trend of the obtained isotherms suggests Cr(VI) sorption on heterogeneous surface. Consequently, the fitting curve representing the Freundlich model, as applicable for an infinite limit sorption, better approximated the experimental points than the Langmuir one. On the other hand, as steams from the value of the Fisher Test (*FT*), mean error (*ME*), and the approximation of standard deviation (*σ*) (Table S2), the best approximation was obtained for the Bilangmuir model. This model, however, highly overestimated the experimentally obtained value of maximum sorption capacity (*q*_e,m_). Thus, one cannot suppose that Cr(VI) uptake is associated with monolayer adsorption on two kinds of active sites. This shall in particular apply where mathematical description of the sorption process does not relate to binding different ionic forms of Cr(VI). At pH_in_ 3, depending on concentration, the monovalent oxyanion of HCrO_4_^−^ (for lower concentration) and/or divalent Cr_2_O_7_^2−^ (for higher concentration) predominate. Hence, in order for the model to represent the Cr(VI) sorption on double-layers SMZs accurately, it should consist of terms of at least two equations relating to ion exchange of Br^-^/HCrO_4_^−^ and Br^-^/Cr_2_O_7_^2−^. Its usage is rather doubtful since the ration of both forms changes along with pH and concentration as the sorption process progresses. Apart from this, such a model should be extended by proposing terms of equation related to other possible binding mechanisms as well.

**Figure 9:**
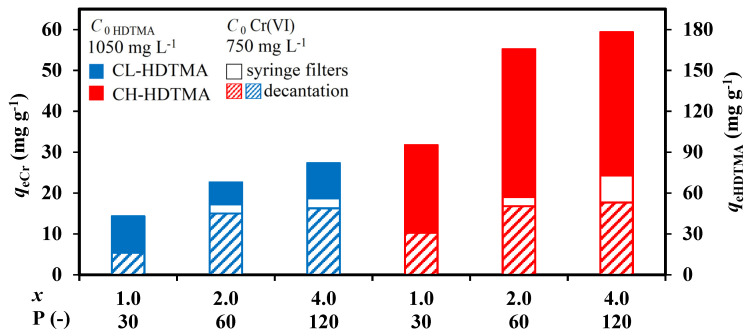
Comparison of efficiency of Cr(VI) sorption on SMZs (*m* = 0.1 g, *C*_0 Cr_ = 750 mg L^−1^, pH 3, *t*_c_ = 24 h).

The stack column graph on Figure 9 summarizes the efficiency of Cr(VI) sorption (pattern bars, equilibrium solution after decantation) in relation to the efficiency of zeolites modification (plain bars blue and red). It should be noted that under the applied conditions of zeolite modification, the ratio of *x*:*B* = 1:30 was maintained constant, which allowed to keep the same rate of organic layers formation for each *x*. To compare quantitatively the efficiency of Cr (VI) sorption on CL-HDTMA and CH-HDTMA, the difference between the scale on the Y axis on the left and on the right side was kept 1:3. The comparison of *q*_eCr(VI)_ and *q*_eHDTMA_ clearly identified that higher efficiency of Cr (VI) sorption was obtained for SMZ with higher HDTMA loading. Furthermore, for all tests the *q*_eCr(VI)_:*q*_eHDTMA_ ratios were much lower in CH-HDTMA material case. It is very likely that HDTMA micelles filled cavities on chabazite external surface becoming not accessible for Cr (VI) ions sorption due to the steric effect. Generally, the ratio between *q*_eCr(VI)_:*q*_eHDTMA_ did not behave in a linear manner for both SMZs. The amount of Cr(VI) uptake by the materials modified with *x* = 2.0 ECEC was up to 3 times higher than by the materials with *x* = 1.0 ECEC. Hence, the predominant mechanism of oxyanions uptake by double-layer zeolites was ion exchange between Br^−^/Cr(VI). Moreover, the very fact that the mono-layer zeolites uptake Cr(VI) indicated participation of other mechanisms as well. Furthermore, higher *q*_eCr(VI)_ obtained for materials modified with *x* = 4.0 than with 2.0 ECEC identified higher amounts of active sites available for chromium sorption resulting from more dense packed double-layer. In order to verify the Cr(VI) complexation by unbounded HDTMA aggregates, some part of equilibrium solution of Cr(VI) after decantation was additionally filtered by MCE 0.2 μm Whatman filters. The Cr content analysis (white plain bars in Figure 9) identified no changes in efficiency of Cr(VI) removal by SMZs modified with *x* = 1.0 ECEC, which confirms the stability of organic monolayer formed. In contrast, an increase of *q*_eCr(VI)_ was obtained for SMZs modified with *x* = 2.0 and 4.0 ECEC. Hence, the equilibrium pH_eq_ rose up to 5.4; it is rather unlikely that HDTMA detachment occurs via the exchange reaction (e.g., HDTMA^+^/H^+^) [24]. According to Zeng et al., Cr(VI) sorption causes disintegration of organic phase and release a loosely bonded HDTMA from the zeolite surface during the mechanical shaking [18]. The released HDTMA can react with Cr (VI) anions giving alkylammonium chromates [Zeolit-HDTMA-HCrO_4_]_n_ and dichromates [(Zeolit-HDTMA)_2_-Cr_2_O_7_]_n_ micelles, which are deposited via Van der Waals interactions with alkylammonium form on the zeolites surface [22]. The extent of the disintegration process is probably lower in case of zeolite modified in column conditions than in batch ones. This directly translates into higher Cr(VI) sorption capacities obtained in comparison to the data presented in literature (Table S3).

The comparison of the ATR spectra of zeolitic materials before and after Cr(VI) sorption (Figure 6) shows a shift of the bands of stretching vibrations of asymmetric and symmetric C-H groups of the alkyl chain, visible in the range of 3050–2800 cm^−1^, to higher wavenumber values, after Cr (VI) sorption. At the same time, the intensity of these peaks decreased. The shift is caused by the replacement of small size Br^−^ ions by large chromium ones, while its size depends on the number of anions retained on the outer SMZ’s surface [31]. In both mono- and double-layer SMZs cases, Cr(VI) sorption weakened the intensity of peaks at 1460 cm^−1^ suggesting that the active sites created by HDTMA amino groups are responsible for Cr (VI) binding. Remarkably, the lack of a shift in the position of the peaks in the 1200–500 cm^−1^ band of the Si-O (Si tetrahedron) and Si-O (Al tetrahedron) proves that Cr (VI) ions did not penetrate into the internal structure of the SMZs.

**Figure 10:**
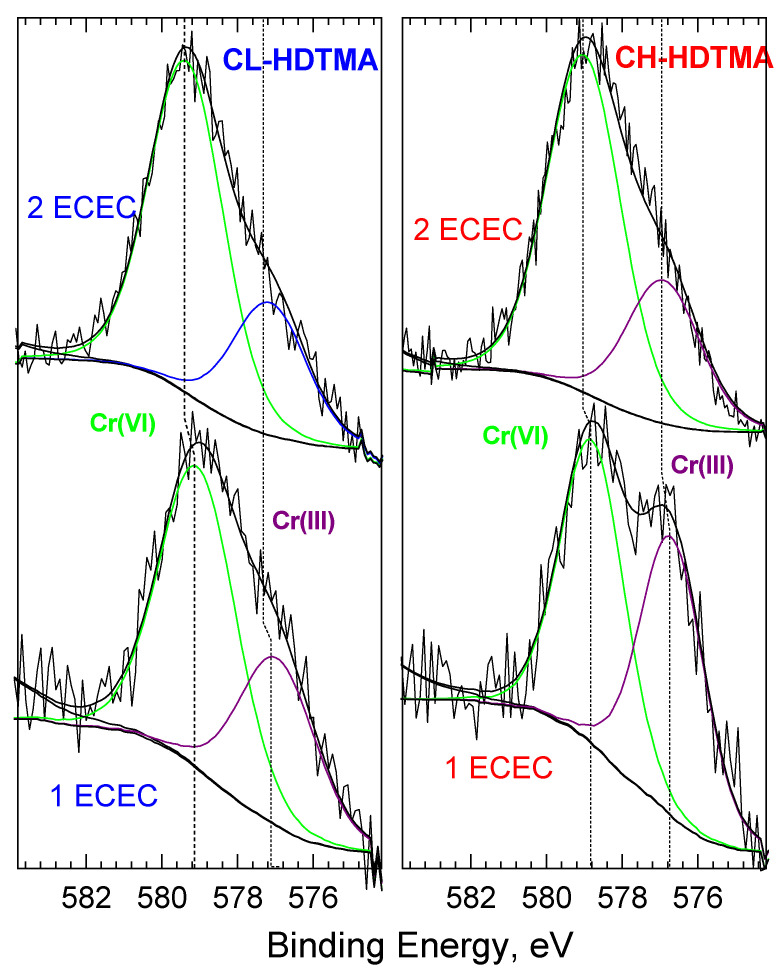
XPS Cr 2p_3/2_ spectra of SMZ after Cr(VI) sorption.

The XPS analysis confirms significantly higher Cr(VI) sorption on double layer SMZs than on mono layer ones (Table 4). As can be seen, there is direct correlation between the amount of Cr(VI) sorbed and N content. Increase of both values was in the range of 0.3–0.4% at., which attests the ion exchange of Cr oxyanions with Br^-^, which is further confirmed by the lack of Br 3d peaks in the double-layer SMZs spectra [32]. A detailed XPS high resolution scans of Cr 2p (Figure 10) reveals two main peaks, corresponding to 2p3/2 and 2p1/2 core levels of chromium. The 2p3/2 peaks located at a binding energy of ∼577 eV correspond to Cr(III) based on values ranging between 577.0 and 578.0 eV for Cr2p3/2 reported for Cr(III)-containing materials. Cr(VI) species like CrO_3_ have higher binding energies; 579.1–580.5 eV [33,34]. Cr(VI) reduction on Fe(II)-bearing minerals and on zerovalent iron was attributed to precipitation of Cr(III)–Fe(III) (hydro)oxides [35,36,37]. This suggests that the Cr(III) compound formed at the surface of the SMZs was Cr(OH)_3_ rather than Cr_2_O_3_. Furthermore, the degree of reduction depends on the zeolitic material and the organic coverage level. In case of mono layer SMZs, the reduction degree was 48% and 35% on CH-HDTMA and on CL-HDTMA, respectively. The identified difference can be explained by higher Fe content in CH tuff (Table 1). The degree of reduction was lower for double layer SMZs and equaled 29.9% and 28% on CH-HDTMA and on CL-HDTMA, respectively. This clearly indicates that the second organic layer additionally limited the Cr(VI) reduction but did not eliminate it.

### 3.3. Real Wastewater Treatment on SMZ

**Figure 11:**
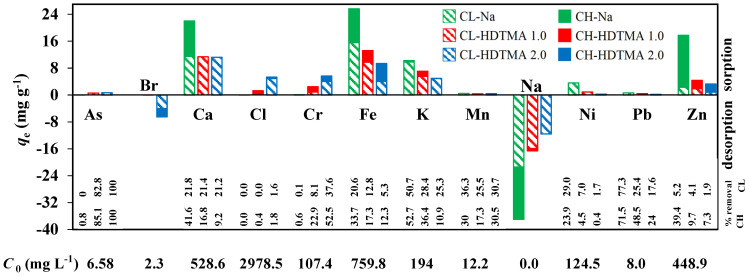
Efficiency of wastewater treatment by SMZs.

Figure 11 shows the results of wastewater analysis provided together with an initial concentration of elements, sorption efficiency (% of removal), and SMZs working capacity (*q*_e_). The values of pH_in_ and conductivity were 1.2 and 82.6 mS cm^−1^, respectively. The comparison of the data obtained identified that elements occurring in cationic forms (Ca, Fe, K, Ni, Mn, Pb, Zn, Cu) were uptaken by all applied zeolitic material with the following efficiency of removal: Na-form > mono-layer SMZs > double-layer SMZs. The amount of Na^+^ cations, which appeared after sorption in the solution decreased accordingly, indicating the cation removal by ion exchange reaction Me^n+^/Na^+^. This confirms that neither mono nor double organic layer prevents cations transport to the zeolitic surface. Elements occurring in wastewater in anionic forms (As, Cl, Cr) were uptaken only by modified zeolites. This fact excluded the presence of Cr(III) in cationic form in the wastewater. The amount of Cl and Cr uptake by mono layer SMZs was more than two times lower than in the double layer case. The presence of Br^−^ ions that appeared only after sorption on double layer SMZs confirms the ion exchange reaction Br^-^/A^n-^ as a predominate mechanism of anions removal. Nevertheless, surface precipitation of insoluble dichromates (e.g., PbCr_2_O_7_) on the SMZ surface one cannot be excluded. Remarkably, although the initial content of Cl^-^ in the wastewater was 29 times greater than that of Cr, and the efficiency of Cl^-^ removal (1.78%) was lower than Cr (52.49%), the capacity of SMZs for both ions was comparable (*q*_e_ = 5.28 and 5.62 mg g^−1^, respectively).

## 4. Summary

Organic modification of natural zeolites in fixed bed column working as a conventional open run unit is limited by the filtration rate and the foaming of surfactant solution. Conducting the process in a sealed circulation unit combined with batching of the surfactant allows not only for the reduction of the time of modification but also for elimination of the surfactant losses. The efficiency of both mono and double layer formation increases with decreasing a volume of batches and increasing the frequency of their dosing. Notably, in case of mono layer coverage, modification proceeded until the surfactant was exhausted, while in the double layer one, until equilibrium of HDTMA concentration in both phases was established. Consequently, modification even with *x* = 4.0 ECEC does not result in formation of compact double organic layer.

The efficiency of Cr(VI) removal on as-prepared SMZs increases with increasing of HDTMA loading and is strongly dependent on the topology of zeolite external surface. In the case of mono layer SMZs, the capacity of CH-HDTMA is almost 100% greater than CL-HDTMA, while in the case of double layer SMZs, the amount of Cr uptake on CH-HDTMA is only 12% greater than on CL-HDTMA. This is a small gain taking into account almost 2.4 times greater amount of HDTMA used for CH zeolite modification. Cr(VI) sorption on SMZ proceeds via a few mechanisms. Ion exchange is the predominant one, but it takes place only if modification resulted in at least partial double layer coverage. Other accompanied processes are complexation on the positive amine heads of the surfactant, surface precipitation in form of insoluble salts, formation of alkylammonium chromates with unreacted HDTMA aggregates, and surface reduction to Cr(III). The fact that the last reaction occurs regardless of the density of organic coverage indicates that the layers are leaky and permeable for ions. This provides the SMZs with the ability to immobilize a wide range of cations via ion exchange or conversion into insoluble salts. The performed test with real wastewater treatment identified higher affinity of SMZs for Cr(VI) than for other anions, even in their large excess. This makes it a promising and cheap material to immobilize pollutants resulting from various industries by creating permeable reactive barriers around landfills. We believe that the proposed methods of zeolites modification enhance the future studies at the pilot-plant scale.

## Figures and Tables

**Table 1:** Chemical compositions (%) of raw zeolite samples obtained from XRF analysis, presented as % *w/w* content of mineral oxides.

Sample	SiO_2_	Al_2_O_3_	CaO	MgO	K_2_O	Na_2_O	Fe_2_O_3_
CL	70.81	12.24	2.784	0.741	3.578	0.357	1.320
CH	58.62	14.55	2.519	0.828	1.253	5.082	3.246

**Table 2:** Textural parameters of raw zeolite samples obtained from N_2_ adsorption at −195.8 °C.

Sample	Area (m^2^ g^−1^)			Pore Volume (mL g^−1^)
	*A*_BET_	*A*_mic_^a^	*A*_ext_	*V*_T_
CL	29.47	8.15	21.33	0.11
CH	340.22	276.23	63.99	0.30

^a^*t*-plot method.

**Table 3:** Exchange capacities of zeolitic materials.

Exchange Capacity	CEC (mval g^−1^)	ECEC (mval g^−1^)	
Analysis	AAS	TOC	CHN
CL	0.938	0.121	0.121
CH	1.657	0.262	0.262

**Table 4:** XPS elemental composition of analyzed materials (% at.) with different time of Ar^+^ etching.

**SMZ**	**C 1s**	**O 1s**	**N 1s**	**Si 2p**	**Al 2p**	**Fe 2p**	**other**	**Br 3d**
CH-1ECEC as rec.	34.1	35.7	1.1	22.6	5.8	0.7	-	
+Ar^+^ 90″	26.3	40.3	1.2	24.4	6.8	1	-	
+Ar^+^ 120″	21.2	43.0	0.9	25.9	7.7	1.3	-	
CL-1ECEC as rec.	25.1	39.3	0.9	26.2	7.6	0.3	-	
+Ar^+^ 90″	21.9	41.7	0.7	27.6	7.3	0.3	-	
+Ar^+^ 120″	19.2	42.5	0.4	29.3	7.7	0.3	-	
CH-2ECEC as rec.	36.1	31.7	1.4	23.7	6.4	0.1	-	0.6
+Ar^+^ 90″	32.6	34.5	0.9	24.8	6.7	0.1	-	0.3
+Ar^+^ 120″	32.7	34.0	0.9	24.8	7.2	0.2	-	0.3
CL-2ECEC as rec.	45.4	29.1	1.5	18.2	4.7	0.5	-	0.6
+Ar^+^ 90″	38.4	33.6	1.3	20.1	5.4	0.8	-	0.5
+Ar^+^ 120″	39.6	32.4	1.4	20.1	5.4	0.7	-	0.5
HDTMA as rec.	34.1	35.7	1.1	22.6	5.8	0.7	-	
	26.3	40.3	1.2	24.4	6.8	1	-	
**SMZ + Cr(VI)**	**C 1s**	**O 1s**	**N 1s**	**Si 2p**	**Al 2p**	**Fe 2p**	**other**	**Br 3d**
CH-1ECEC + Cr as rec.	36.3	34.1	1.3	20.7	5.2	0.6	1.5	0.4
+Ar^+^ 90″	26.6	40.0	0.9	23.6	6.4	0.9	1.3	0.4
+Ar^+^ 120″	26.6	39.6	0.9	23.7	6.8	0.9	1.0	0.5
CL-1ECEC + Cr as rec.	46.6	27.8	1.6	17.3	4.3	0.3	1.3	0.7
+Ar^+^ 90″	37.1	34.5	1.2	19.7	5.1	0.6	1.0	0.8
+Ar^+^ 120″	36.6	33.5	1.2	20.5	5.8	0.7	0.8	0.9
CH-2ECEC + Cr as rec.	30.5	36.2	1	24.3	5.9	0.1	1.6	0.4
+Ar^+^ 90″	20.8	42.1	0.4	26.9	7.4	0.2	1.34	0.5
+Ar^+^ 120″	19.1	42	0.7	28.9	7.5	0.2	1.23	0.5
CL-2ECEC + Cr as rec.	38.8	30.8	1.4	21.4	5.9	0.03	1.1	0.7
+Ar^+^ 90″	31.4	35.9	1.1	23.9	6.2	0.07	0.8	0.6
+Ar^+^ 120″	32.5	35.1	0.8	24.0	6.2	0.10	0.6	0.6

## Data Availability

Data sharing is not applicable to this article.

## Supplementary Materials

The following are available online at https://www.mdpi.com/article/10.3390/ma14227061/s1, Figure S1: The experimental setup, Figure S2: The schedule of zeolites modification, Table S1: Surfactants used for natural zeolite modification, Table S2: Fitting parameters of equilibrium models, Table S3: The adsorption capacities of HDTMA-modified clinoptilolite and chabazite.
