# Supplementary material for: Fixed-Bed Modification of Zeolitic Tuffs and Their Application for Cr(VI) Removal
Source: Materials (Basel). 2021 Nov 21;14(22):7061. doi: 10.3390/ma14227061 (PMC8621222; doi:10.3390/ma14227061)
Supplement: Supplementary file 1 [file materials-14-07061-s001.zip › materials-1435657-SI.pdf]

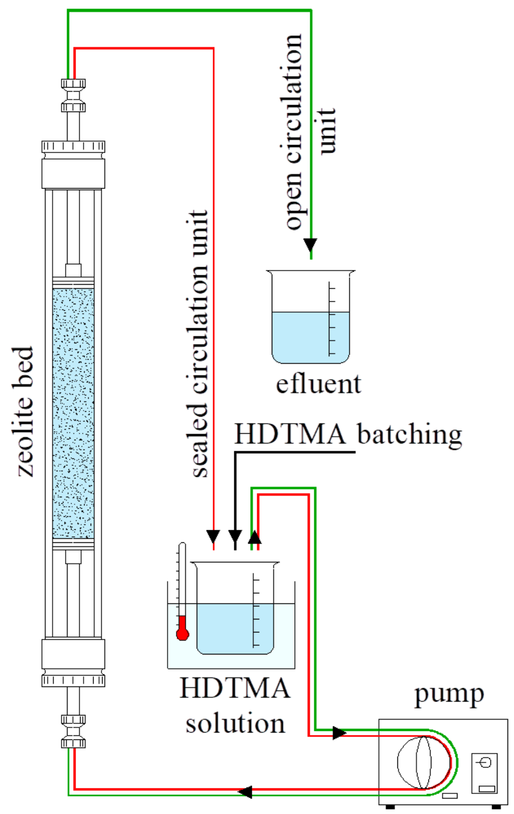

Figure S1. The experimental set up.

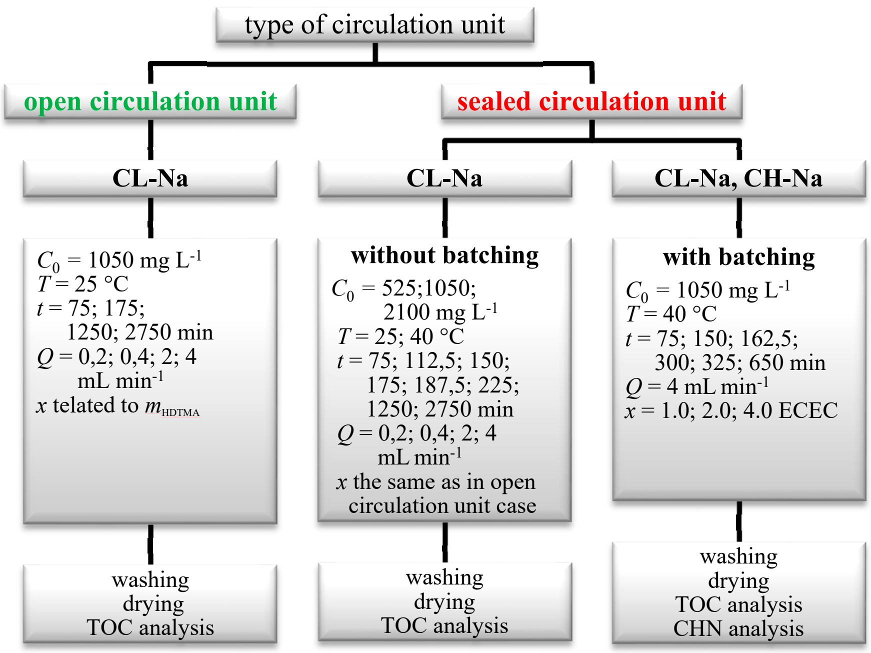

Figure S2. The schedule of zeolites modification.

Table S1. Surfactants used for natural zeolite modification.

Surfactant	Molecular formula	Molecular structure
BDTDA<br>benzyldimethyltetradecyl<br>ammonium	$C_{23}H_{42}N^+$	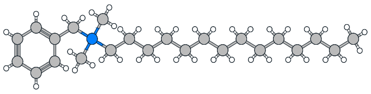
BTEA<br>benzyltriethylammonium	$C_{13}H_{22}N^+$	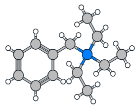
CP<br>cetylpyridine	$C_{21}H_{38}N^+$	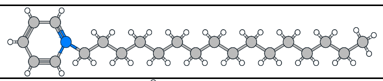
DBDMA<br>dibenzyldimethyl-<br>ammonium	$C_{16}H_{20}N^+$	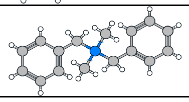
DDMIM<br>1-dodecyl-3-methyl<br>imidazolium	$C_{16}H_{31}N_2^+$	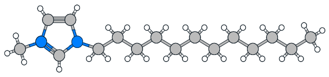
DMC<br>dodecylamine	$C_{12}H_{27}N^+$	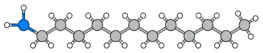
DODA<br>dimethyldioctadecyl-<br>ammonium	$C_{38}H_{80}N^+$	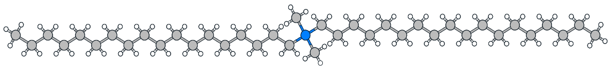
EHDDMA<br>ethylhexadecyldimethyl-<br>ammonium	$C_{20}H_{44}N^+$	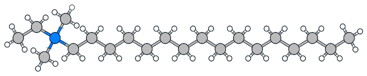
HDMIM<br>1-hexadecyl-<br>3-methylimidazolium	$C_{20}H_{39}N_2^+$	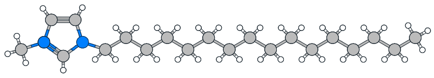
HDP<br>hexadecylpyridinium	$C_{65}H_{126}N^+$	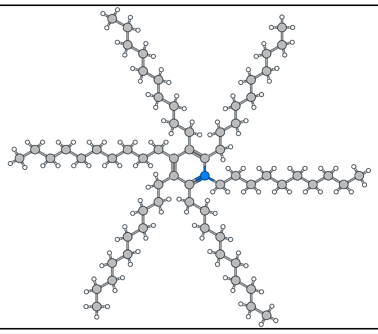
HDTMA<br>hexadecyltrimethyl-<br>ammonium	$C_{19}H_{42}N^+$	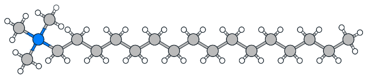
HMC<br>hexadecylamine	$C_{16}H_{35}N^+$	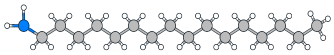
ODA<br>octadecyl amine	$C_{20}H_{43}N^+$	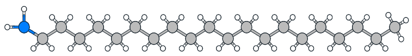
ODMBA<br>octadecylbenzyldimethyl-<br>ammonium	$C_{27}H_{50}N^+$	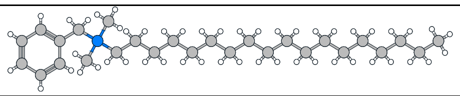
ODTMA<br>octadecyltrimethyl-<br>ammonium	$C_{21}H_{46}N^+$	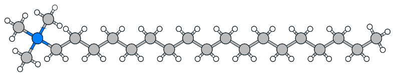
OMC<br>oleylamine	$C_{18}H_{37}N^+$	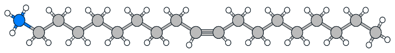

PANI polyaniline	$(C_6H_5N^+)_n$	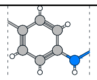 n
	$(C_6H_5N^+)_n$	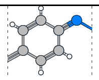 n
	$[(C_6H_5N^+)_2 (C_6H_4N^+)_2]_n$	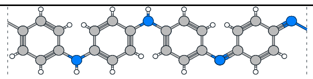 n
PEI poliethylimine	$C_2H_5N^+$	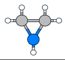
PHMG polyhexamethylene guanidine	$(C_7H_{15}N_3^+)_n$	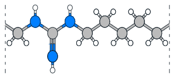 n
TEA tetraethyl ammonium	$C_8H_{20}N^+$	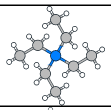
TMA tetramethyl ammonium	$C_4H_{12}N^+$	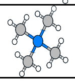

Table S2. Fitting parameters of equilibrium models.

Model	K_1 (L mg^{-1})	K_2 (L mg^{-1})	q_{m1} (mg g^{-1})	q_{m2} (mg g^{-1})	n	TF	ME (%)	σ
x = 1.0 $q_{e,m}$= 5.39 mg g^{-1}								
Langmuir	0.0006	-	19.2	-	-	3115	4.4	0.03
Freundlich	0.0233	-	-	-	1.2	1368	5.5	0.04
Bi–Langmuir	0.0005	0.0394	22.09	0.11	-	10235	0.9	0.02
x = 2.0 $q_{e,m}$= 14.97 mg g^{-1}								
Langmuir	0.0079	-	15.7	-	-	8	28.3	1.37
Freundlich	1.1051	-	-	-	2.5	59	8.1	0.50
Bi–Langmuir	0.2533	0.0004	4.8	54.91	-	379	6.2	0.20
x = 1.0 $q_{e,m}$= 10.25 mg g^{-1}								
Langmuir	0.0022	-	16.5	-	-	54	22.8	0.45
Freundlich	0.1671	-	-	-	1.6	427	8.0	0.16
Bi– Langmuir	0.0003	0.0922	51.4	1.60	-	2023	3.0	0.07
x = 2.0 $q_{e,m}$= 16.77 mg g^{-1}								
Langmuir	0.0224	-	15.4	-	-	15	39.6	1.39
Freundlich	2.2529	-	-	-	3.2	271	14.8	0.32
Bi–Langmuir	0.0021	0.5660	19.3	5.65	-	102	25.2	0.53

Table S3. The adsorption capacities of HDTMA modified clinoptilolite and chabazite.

Surfactant	Zeolitic material	q_{eCr} (mg g^{-1})	pH_{init}	Literature
HDTMA-Br	Clinoptilolite	~ 0.90	3	[38]
HDTMA-Br	Clinoptilolite	1.88	3	[39]
HDTMA-Br	Clinoptilolite	~ 3.50	3	[18]
HDTMA-Br	Clinoptilolite	13.62	3	[40]
HDTMA-Br	Clinoptilolite	14,97	3	this work
HDTMA-Br	Clinoptilolite	2.18	4	[41]
HDTMA-Br	Clinoptilolite	5.82	4	[31]
HDTMA-Br	Clinoptilolite	7.8	4	[17]
HDTMA-Br	Clinoptilolite	17.73	4	[42]
HDTMA-Br	Clinoptilolite	4.10	6	[43]
HDTMA-Br	Clinoptilolite	~ 0.19	6.5	[44]
HDTMA-Br	Clinoptilolite	~ 0.57	7.4	[45]
HDTMA-Br	Clinoptilolite	~ 0.52	7.7	[10]
HDTMA-Br	Chabazite	~ 7.30	3	[18]
HDTMA-Br	Chabazite	16,77	3	this work
HDTMA-Br	Chabazite	10.30	4	[31]
HDTMA-Br	Chabazite	2.60	6	[22]

[38] Salgado-Gómez N., Macedo-Miranda M.G., Olguín M.T., 2014. Chromium VI adsorption from sodium chromate and potassium dichromate aqueous systems by hexadecyltrimethylammonium-modified zeolite-rich tuff. Appl Clay Sci 95, 197-204 https://doi.org/10.1016/j.clay.2014.04.013
[39] Chojnacka M., Warchoł J., Sobolewska P., 2018. Effect of the pH on the Cr(VI) Sorption on HDTMA-zeolites. J Environ Sci Eng A 7, 261-267 https://doi.org/10.17265/2162-5298/2018.07.001
[40] Bajda T., Kłapyta Z., 2013. Adsorption of chromate from aqueous solutions by HDTMA-modified clinoptilolite, glauconite and montmorillonite. Appl Clay Sci 86, 169-173 https://doi.org/10.1016/j.clay.2013.10.005
[41] Warchoł J., Misaelides P., Petrus R., Zamboulis D., 2006. Preparation and application of organo-modified zeolitic material in the removal of chromates and iodides. J Hazard Mater B137, 1410-1416 https://doi.org/10.1016/j.jhazmat.2006.04.028
[42] Mozgawa W., Król M., Bajda T., 2011. IR spectra in the studies of anion sorption on natural sorbents. J Mol Struct 993, 109-114 https://doi.org/10.1016/j.molstruc.2010.11.070
[43] Leyva-Ramos R., Jacobo-Azuara A., Diaz-Flores P., Guerrero-Coronado R., Mendoza-Barron J., Berber-Mendoza M., 2008. Adsorption of chromium(VI) from an aqueous solution on a surfactant-modified zeolite. Colloid Surface A 330, 35-41 https://doi.org/10.1016/j.colsurfa.2008.07.025
[44] Haggerty G.M., Bowman R.S., 1994. Sorption of chromate and other inorganic anions by organo-zeolite. Environ Sci Technol 28, 452-458 https://doi.org/10.1021/es00052a017
[45] Ghiaci M., Kia R., Abbaspur A., Seyedeyn -Azad F., 2004. Adsorption of chromate by surfactant modified zeolites and MCM-41 molecular sieve. Sep Purif Technol 40, 285-295 https://doi.org/10.1016/j.seppur.2004.03.009
